# Does the practice of mindfulness reduce somatic symptoms and COVID-19-related anxiety? A community-based survey

**DOI:** 10.3389/fpsyg.2022.996559

**Published:** 2022-12-09

**Authors:** Noemi Micheli, Piero Porcelli, Marion Barrault-Couchouron, Cécile Dantzer

**Affiliations:** ^1^Univertité de Bordeaux, LabPsy UR 4139, Bordeaux, France; ^2^Department of Psychological, Health and Territorial Sciences, “G. d’Annunzio” University of Chieti-Pescara, Chieti, Italy; ^3^Bordeaux Population Health, Inserm U1219, Université de Bordeaux, Bordeaux, France

**Keywords:** mindfulness practice, dispositional mindfulness, COVID-19 anxiety, somatic symptoms, emotional regulation strategies

## Abstract

**Objective:**

Since the beginning of COVID-19 pandemic, several studies have shown an increase of psychological distress in the general population. Previous research demonstrated that high levels of anxiety are associated with reporting more somatic symptoms. The ability to adaptively regulate emotions is essential to deal with stressful situations, and it is one of the main components of mindfulness practice. The aim of the present study was to document the effect of mindfulness practice on somatic symptoms and psychological distress in the context of COVID-19 pandemic.

**Methods:**

The study has a descriptive cross-sectional design. During the second wave of COVID-19 pandemic, between November 2020 and January 2021 participants living in France responded to an online survey on the impact of COVID-19 on psychological distress and physical health. The questionnaire included the assessment of COVID-19-related anxiety, mindfulness practice and experience, dispositional mindfulness, somatization, depression, generalized anxiety, and emotion regulation.

**Results:**

A total of 569 people (mean age = 39.8 years, 90% women) were included in the study. COVID-19 related anxiety was associated with higher levels of somatic symptoms, generalized anxiety, and depression. About half of the sample (*n* = 318, 56%) reported moderate to severe somatic symptoms that were associated with higher levels of depression and anxiety, lower levels of dispositional mindfulness and to the use of maladaptive emotion regulation strategies. Overall, 164 subjects (28.8%) reported practicing meditation. No differences were found in dispositional mindfulness (MAAS score) between beginners and advanced practitioners, regardless of the type, years, frequency, and length of practice. Participants with less experience in mindfulness practice reported a significant higher number of somatic symptoms than non-practitioners and a higher use of rumination. Moreover, mindfulness experience was associated with the use of more adaptive emotion regulation strategies.

**Conclusion:**

Mindfulness meditation has been promoted as a practice enhancing well-being and helping to cope with the psychological impact of stressful events. However, in a distressing situation as COVID-19 pandemic, a limited experience in mindfulness practices might result in the development or endurance of somatic symptoms. Adequate training and a focus on mindful acceptance, may contribute to enhance the effectiveness of mindfulness practice.

## Introduction

In 2019, the World has been stroked by the spreading of the new SARS-CoV-2 coronavirus, bringing the World Health Organization to officially declare the COVID-19 outbreak as a pandemic on March 11, 2020. Population’s daily life has been altered by unprecedented public health measures, causing people to experience social isolation, adverse media exposure, worry about significant others, and limited access to health services (Jernigan, 2020; Cruz et al., 2021; Talic et al., 2021). As a result, the pandemic has created not only the “perfect storm” for an augmentation of anxiety and depression (Barzilay et al., 2020; Lakhan et al., 2020; Prete et al., 2020; Salari et al., 2020; Boden et al., 2021), which increased through the two lockdown periods (Lu et al., 2022), but also of persistent somatic symptoms (Bruno et al., 2020; Shevlin et al., 2020; Willis and Chalder, 2021).

Persistent somatic symptoms, also defined as medically unexplained symptoms or functional somatic symptoms, can result from a tendency to experience and communicate psychological distress in the form of physical symptoms and to seek medical help for them (Lipowski, 1988; Roenneberg et al., 2019). Somatic Symptom Disorder (SSD) is the corresponding diagnosis in the fifth edition of the Diagnostic and Statistical Manual of Mental Disorders (DSM-5), and refers to persistent (6 months or more) and clinically significant somatic complaints accompanied with excessive health-related thoughts, feelings, and behaviors, regardless of the functional vs. organic nature of the symptoms (American Psychiatric Association, 2013). The most common symptoms include pain, fatigue, and disturbances in the organ functions (Witthöft et al., 2016). The underlying mechanisms of the development of somatic symptoms are still not fully understood (Groen et al., 2021), but research has shown that they are associated with at least a 2-fold increase in the likelihood of having a depressive and/or anxiety disorder (Kroenke et al., 1997; Janssens et al., 2010; van Boven et al., 2011; Groen et al., 2021). This might due to the fact that high-state anxiety and depression can induce physiological changes, an intensified attention to the body and an alteration of the cognitive processing of physical sensations, representing important precipitating factors (Mayou and Farmer, 2002; Janssens et al., 2010; Mallorquí-bagué et al., 2016). Indeed, the attention to bodily sensations is usually high in subjects who report somatic symptoms (Mirams et al., 2013; Mehling, 2016) and tend to interpret physical sensations as a sign of illness (Duddu et al., 2003; Rief et al., 2004). In a distressing situation such as COVID-19 pandemic, during which people had to be particularly attentive to any signs of virus infection, these attribution and regulation tendencies might have been heightened. In fact, while the prevalence of persistent somatic symptom in the general population ranges between 6.7 and 17.4%, it has been found to be 45.9% at the peak of the pandemic (Ran et al., 2020), which is similar to the one usually recorded in primary care patients (Löwe et al., 2022).

Studies assessing the practice of mindfulness meditation during COVID-19 showed positive results on the psychological distress of the population, suggesting that mindfulness meditation might be a viable intervention for the impact of the pandemic on mental health (Matiz et al., 2020; Bursky et al., 2021; Zhu et al., 2021). Mindfulness meditation, training the propensity to attend to and accept the present moment experiences (Brown and Ryan, 2003; Baer and Krietemeyer, 2006), facilitates the positive reappraisal of stressful life events, decreasing maladaptive coping cognitive processes known to prolong the experiences of psychological distress (Nolen-Hoeksema, 2000; Jain et al., 2007; Garland et al., 2011). This increase in emotion regulation is crucial in patients with somatic complaints as a lack of emotional awareness and an overuse of maladaptive emotion regulation strategies are considered basic psychological liabilities of Somatic Symptom Disorder (Waller and Scheidt, 2004; Garnefski et al., 2017; Okur Güney et al., 2019). Previous research has shown that mindfulness meditation can have a positive impact on some aspects of somatization, such as symptom severity and pain intensity, and on comorbid anxiety and depression (Hoge et al., 2013; Lakhan and Schofield, 2013; Aucoin et al., 2014; Hilton et al., 2017; Hazlett-Stevens, 2018; Billones et al., 2020). However, the body of evidence prevents strong conclusions, as available studies are mostly based on mindfulness interventions, in which social determinants, such as the relationship with the instructor and the group, have been found to be stronger predictors than specific mindfulness practice-related factors on psychological outcomes (Canby et al., 2021). Furthermore, mindfulness can be a state, a training, or a disposition that can be promoted through training, but in the evidence, a clear distinction is not always made between intentional mindfulness meditation and dispositional mindfulness (Rau and Williams, 2016; Wheeler et al., 2016; Tang et al., 2017). Dispositional mindfulness has protective effects against psychological distress (Tomlinson et al., 2018). However, different levels of dispositional mindfulness have been observed in the population, independently of mindfulness practice (Brown et al., 2007). Intentional mindfulness meditation, instead, can induce subjects into temporary states of mindfulness and bring to modifications in personality traits following a longer period of regular practice (Shapiro et al., 2011; Nyklíček et al., 2013; Tang et al., 2015, 2017). In fact, a longer experience in meditation is associated with higher levels of dispositional mindfulness (Josefsson et al., 2011; Tomlinson et al., 2018), and with a reduction of maladaptive emotion regulation strategies (Schlosser et al., 2020).

To our best knowledge, no other study has looked at the effect of intentional mindfulness meditation on somatization and psychological distress during COVID-19 pandemic. The purpose of this observational study is to fill this gap, taking into account dispositional mindfulness, meditation experience, and emotion regulation. Three hypotheses were formulated. First, dispositional mindfulness would be associated with lower somatic symptom, psychological distress, and COVID-19-related anxiety. Second, a longer experience in mindfulness meditation, enhancing dispositional mindfulness and emotion regulation, would be associated with less somatic symptoms and psychological distress. Third, practitioners with less experience would report more somatic symptoms and a greater use of maladaptive emotion regulation strategies that those with more experience.

## Materials and methods

During the worldwide second wave of pandemic, from November 24 2020 to January 29 2021, 790 participants living in France responded to an online survey about the impact of COVID-19 on emotions and physical health. Participants were recruited through free social network advertisement. To maximize ecological validity, the only inclusion criterion was being at least 18 years old. The study followed the local ethics protocol: all subjects provided informed consent, through an online form, in accordance with the Declaration of Helsinki.

### Participants

569 (53.48%) subjects provided at least 50% of data and were included in the study. The mean age of the total sample was 39.8 years (SD = 14.5, range = 18–89). Most of the participants were female (*n* = 512, 90%), with a high level of education or university degrees (54.3%), employed (60.5%), and living with their families or partners (78.4%). A quarter of the sample were infected by the SARS-CoV-2 (*n* = 139, 24.4%), and half of the participants reported COVID-19 infections among close friends or relatives. Finally, 17.6% of the population self-reported the presence of psychological problems, and 25% declared having a chronic illness (see Supplementary Table 1 for detailed descriptive statistics in Supplementary material).

### Measures

Socio-demographic and COVID-19-related information (e.g., having contracted the virus and the presence/absence of positive cases among relatives or friends) were among the data collected.

As in other studies, COVID-19-related anxiety was assessed through the question: “How anxious are you about the coronavirus COVID-19 pandemic?” (Bruno et al., 2020; Shevlin et al., 2020). Participants were provided with an electronic visual analog scale to indicate their degree of anxiety, ranging from 0 to 10, with higher scores reflecting higher levels of COVID-19-related anxiety.

Mindfulness practice was determined through six questions asking participants if they had a regular mindfulness practice, years of practice, weekly frequency, usual practice length, and amount of practice in the last month compared to usual and type of mindfulness exercise. The variable years of practice was used to determine mindfulness experience as it was judged to be less subject to recall bias and measurement error than frequency and session length (see Schlosser et al., 2020). Subjects practicing for less than 2 years were categorized as “beginners,” while people reporting a longer meditation experience (more than 2 years) have been classified as “advanced” (Haimerl and Valentine, 2001).

Dispositional mindfulness was assessed with the Mindful Attention Awareness Scale (MAAS; Brown and Ryan, 2003), a 15 items scale to measure mindfulness as a trait. Each item is scored using a six-point Likert scale ranging from 1 (“almost always”) to 6 (“almost never”). The total score is calculated by the mean of the 15 items, with higher scores indicating greater dispositional mindfulness. The total score reliability in the current sample was high (*α* = 0.89).

Somatic symptoms were evaluated using the Patient Health Questionnaire (PHQ-15; Kroenke et al., 2001), a self-report measure that assessing physical health problems in the last 2 weeks. Items are rated on a three-point Likert scale ranging from 0 (“not bothered at all”) to 2 (“bothered a lot”). The total PHQ-15 score ranges from 0 to 30. Previous studies attest that PHQ-15 produces reliable and valid scores (Hinz et al., 2017). The total score reliability in the current sample was high (*α* = 0.80).

Psychological distress was measured with the Generalized Anxiety Disorder 7-item Scale (GAD-7; Spitzer et al., 2006) and the Patient Health Questionnaire-8 (PHQ-8; Kroenke et al., 2009). The PHQ-8 includes eight of the nine criteria on which the DSM-IV diagnosis of depressive disorders is based (American Psychiatric Association, 1994). As in previous studies, the ninth criteria assessing suicidal and self-injurious thoughts was omitted as researchers would not be able to provide adequate intervention due to the online design of the study and its deletion has only a minor effect on scoring (Kroenke and Spitzer, 2002; Kroenke et al., 2009). Higher scores represent increased severity of anxiety and depressive symptoms, and the cut-off score of ≥10 was used to identify a moderate level of anxiety and depressive symptoms (Kroenke et al., 2009, 2010; Williams, 2014). The reliability in the current sample for both scales was high (GAD-7: *α* = 0.91; PHQ-8: *α* = 0.87).

Emotion regulation was assessed with the Cognitive Emotion Regulation Questionnaire (CERQ; Garnefski et al., 2001). The CERQ is a 36-item questionnaire which uses a five-point Likert scale to assess emotion regulation strategies that subjects use in response to the experience of stressful events. The final scores are divided in nine strategy subscales, grouped into adaptive (CERQ_A: acceptance, positive refocusing, refocus on planning, positive reappraisal, and putting into perspective), and maladaptive strategies (CERQ_M: self-blame, rumination, catastrophizing, and blaming others; Garnefski et al., 2001; Aldao and Nolen-Hoeksema, 2010). In the current study, the Cronbach’s alpha ranged from 0.66 to 0.87 for each strategy.

### Statistical analyses

Analyses were performed using R version 3.0 (R Core Team, 2013). The R code used for statistical analyses is available in the Supplementary material. Multiple imputation by chained equations (MICE) using a predictive mean matching method has been performed on the 3.8% of data, representing missing values in the mandatory questions of the survey. Descriptive statistics were used to describe the socio-demographic characteristics of the respondents. Measurement data were expressed as the mean ± SD, while independent sample *t*-tests were used for group comparisons (practitioners vs. non-practitioners). Pearson’s correlations were used to assess the association between COVID-19 related anxiety, generalized anxiety, depression, somatization, emotion regulation, and dispositional mindfulness. Given that the relationship between meditation experience and the variables of interest was not assumed to be linear, meditation experience was categorized into three groups: non-practitioners, beginners (mindfulness practice ≤2 years) and advanced (mindfulness practice >2 years). For the group comparisons between non-practitioners, beginner, and advanced practitioners, one-way ANOVA was used. The values of *p* < 0.05 were considered statistically significant. Multiple comparisons were performed by Tukey–Kramer Test for *post hoc* analysis to locate the significant group-by-group differences.

## Results

Descriptive statistics of responders’ socio-demographic characteristics and comparisons between practitioners and non-practitioners are available in the Supplementary Table 1. Overall, there were no main socio-demographic differences between the group of practitioners and non-practitioners except for age and educational level (practitioners were younger and more educated than non-practitioners).

### Dispositional mindfulness, somatic, and psychological distress

Table 1 shows zero-order associations between COVID-19-related variables, somatic and psychological symptoms. As expected, large associations (*r* > −0.50) were found between psychological distress (i.e., anxiety and depression scores) and somatic symptoms. Dispositional mindfulness showed strong and negative correlations with psychological distress [*r*(567) = −0.56, *p* < 0.001 for both anxiety and depression], somatic symptoms [*r*(567) = −0.46, *p* < 0.001], and a small and negative correlation with COVID-19 related anxiety [*r*(567) = −0.13, *p* < 0.001]. Moreover, dispositional mindfulness was negatively associated with maladaptive emotion regulation, [*r*(567) = −0.37, *p* < 0.001].

**Table 1:** Associations between COVID-19-related factors, somatic and psychological variables.

	*COVID-19 anxiety*	*1*	*2*	*3*	*4*	*5*	*6*	*7*	*8*	*9*
*1. Chronic Illness*	0.004 (*0.931*)									
*2. Psychological problems*	0.089 (*0.033*)	−0.001 (*0.975*)								
*3. Coronavirus infection*	0.134 (*0.001*)	0.015 (*0.723*)	−0.046 (*0.270*)							
*4. Covid proxy*	−0.048 (*0.255*)	0.001 (*0.981*)	−0.126 (*0.003*)	0.050 (*0.229*)						
*5. PHQ-15*	0.234 (*<0.001*)	0.136 (*0.001*)	0.093 (*0.027*)	0.230 (*<0.001*)	−0.040 (*0.338*)					
*6. CERQ Maladaptive*	0.181 (*<0.001*)	0.015 (*0.715*)	0.200 (*<0.001*)	0.089 (*0.033*)	−0.028 (*0.512*)	0.296 (*<0.001*)				
*7. CERQ Adaptive*	−0.066 (*0.118*)	−0.035 (*0.410*)	−0.121 (*0.004*)	−0.025 (*0.544*)	0.047 (*0.267*)	−0.073 (*0.081*)	0.028 (*0.503*)			
*8. PHQ-8*	0.232 (*<0.001*)	0.036 (*0.386*)	0.222 (*<0.001*)	0.155 (*<0.001*)	−0.047 (*0.265*)	0.589 (*<0.001*)	0.468 (*<0.001*)	−0.121 (*0.004*)		
*9. GAD-7*	0.316 (*<0.001*)	0.006 (*0.895*)	0.160 (*<0.001*)	0.096 (*0.023*)	−0.063 (*0.133*)	0.575 (*<0.001*)	0.473 (*<0.001*)	−0.114 (*0.007*)	0.731 (*<0.001*)	
*MAAS*	−0.138 (*0.001*)	−0.039 (*0.358*)	−0.118 (*0.005*)	−0.039 (*0.357*)	0.019 (*0.649*)	−0.462 (*<0.001*)	−0.378 (*<0.001*)	0.069 (*0.101*)	−0.565 (*<0.001*)	−0.567 (*<0.001*)

Computed correlation used Pearson-method with list wise-deletion. PHQ-15, Patient Health Questionnaire—Somatic Symptom Severity Scale; CERQ, Cognitive Emotion Regulation Questionnaire; PHQ-8, Patient Health Questionnaire for Depression; GAD-7, Generalized Anxiety Disorder; and MAAS, Mindful Attention Awareness Scale.

### The effect of mindfulness practice and experience on somatic symptoms and psychological distress

Mindfulness meditation was practiced by 28.8% of the population (*n* = 164). The years of experience in mindfulness practice as well as the amount of practice were quite homogenous in the sample of practitioners. Most practitioners practiced either for 10 min (*n* = 67) or between 10 and 30 min (*n* = 67), but a smaller part had a length of practice of more than 30 min for each session (*n* = 30). In total, 87 (15.3%) subjects were categorized as beginners (≤2 years of practice) and 77 people (13.5%) as advanced (>2 years of practice). A significant difference was found in the type of practice (*p* = 0.003; Table 2).

**Table 2:** Descriptive statistics of mindfulness practice.

	Overall (*N* = 569)	Beginners (*N* = 87)	Advanced (*N* = 77)	*X^2^*	Value of *p*	*V*
**Years of practice**						
Less than 1 year	40 (7%)	40 (46%)	-			
Between 1 and 2 years	47 (8.3%)	47 (54%)	-			
Between 3 and 4 years	35 (6.2%)	-	35 (45.5%)			
More than 4 years	42 (7.4%)	-	42 (54.5%)			
**Practice per week**						
Less than once a week	40 (7.0%)	21 (24.1%)	19 (24.7%)	7.597	0.1	0.215
Once a week	36 (6.3%)	25 (28.7%)	11 (14.3%)			
Between 2 and 3 times	55 (9.7%)	29 (33.3%)	26 (33.8%)			
Between 4 and 5 times	16 (2.8%)	6 (6.9%)	10 (13.0%)			
More than six times	17 (3.0%)	6 (6.9%)	11 (14.3%)			
**Length of practice**						
10 min	67 (11.8%)	39 (44.8%)	28 (36.4%)	1.215	0.5	0.086
Between 10 and 30 min	67 (11.8%)	33 (37.9%)	34 (44.2%)			
More than 30 min	30 (5.3%)	15 (17.2%)	15 (19.5%)			
**Type of practice**						
Yoga	27 (4.7%)	21 (24.1%)	6 (7.8%)	17.67	0.003	0.328
Mindfulness meditation	26 (4.6%)	12 (13.8%)	14 (18.2%)			
Breathing exercises	48 (8.4%)	29 (33.3%)	19 (24.7%)			
Yoga and meditation	31 (5.4%)	11 (12.6%)	20 (26%)			
Meditation and breathing exercises	27 (4.7%)	14 (16.1%)	13 (16.9%)			
Other practices	5 (0.9%)	0 (0%)	5 (6.5%)			

One-way ANOVA comparison showed no differences between non-practitioners, beginners, and advanced practitioners concerning dispositional mindfulness (Table 3). Moreover, no differences were found among the three groups concerning depression, anxiety, and COVID-19 related anxiety. However, a significant difference was found between groups in the levels of somatic symptoms [*F*(2, 566) = 4.50, *p* = 0.01, *η^2^* = 0.01]. Tukey–Kramer *post hoc* tests showed that beginners reported significantly more somatic symptoms (*M* = 12.2) than non-practitioners (*M* = 10.3, *p* = 0.008).

**Table 3:** Descriptive statistics of somatic and psychological variables in the population and according to practice group.

	Total (*N* = 569)	Non-practitioners (NP) (*N* = 405)	Practitioners		*F*	Value of *p*	η^2^	*Post hoc* test (Tukey)
			Beginners (BE) (*N* = 87)	Advanced (AD) (*N* = 77)				
**COVID-19 anxiety**								
Mean ± SD [Min, Max]	4.24 ± 2.86 [0, 10]	4.20 ± 2.87	4.77 ± 2.68	3.87 ± 2.97	2.19	0.11	0.007	-
**Somatic symptoms (PHQ-15)**								
Mean ± SD [Min, Max]	10.7 ± 5.37 [0, 30]	10.3 ± 5.32	12.2 ± 4.69	10.9 ± 6.09	4.50	0.01	0.015	NP < BE
Score 0–4	74 (13%)	57 (14%)	3 (3%)	14 (18%)				
Score 5–9 (mild)	177 (31%)	136 (34%)	22 (25%)	19 (25%)				
Score 10–14 (moderate)	183 (32%)	124 (31%)	36 (41%)	23 (30%)				
Score ≥ 15 (severe)	135 (24%)	88 (22%)	26 (30%)	21 (27%)				
PHQ-15 score ≥ 10	318 (56%)	212 (52%)	62 (71%)	44 (57%)				
**Depression (PHQ-8)**								
Mean ± SD [Min, Max]	9.51 ± 6.14 [0, 24]	9.34 ± 6.10	10.3 ± 6.02	9.48 ± 6.47	0.91	0.40	0.003	-
Score 0–4 (none)	137 (24%)	99 (24%)	16 (18%)	22 (29%)				
Score 5–9 (mild)	173 (30%)	126 (31%)	27 (31%)	20 (26%)				
Score 10–14 (moderate)	136 (24%)	97 (24%)	22 (25%)	17 (22%)				
Score 15–19 (moderate severe)	81 (14%)	55 (14%)	13 (15%)	13 (17%)				
Score 20–24 (severe)	42 (7%)	28 (7%)	9 (10%)	5 (6%)				
PHQ-8 score ≥ 10	259 (46%)	180 (44%)	44 (51%)	35 (45%)				
**Anxiety (GAD-7)**								
Mean ± SD [Min, Max]	8.42 ± 6.05 [0, 21]	8.40 ± 6.09	8.75 ± 5.63	8.14 ± 6.32	0.21	0.80	0.000	-
GAD-7 score ≥ 10	205 (36%)	143 (35%)	33 (38%)	29 (38%)				
**Depression and anxiety**	173 (30%)	120 (30%)	27 (31%)	26 (34%)				
**CERQ adaptive**								
Mean ± SD [Min, Max]	61.2 ± 14.8 [20, 100]	59.9 ± 14.7	61.8 ± 14.4	66.9 ± 14.3	7.32	<0.001	0.025	NP < AD
**CERQ maladaptive**								
Mean ± SD [Min, Max]	36.8 ± 10.4 [16, 76]	36.8 ± 10.8	38.4 ± 9.47	34.9 ± 9.13	2.27	0.10	0.007	-
**Dispositional mindfulness (MAAS)**								
Mean ± SD [Min, Max]	3.75 ± 0.87 [1, 5.93]	3.77 ± 0.85	3.74 ± 0.86	3.68 ± 0.93	0.35	0.70	0.001	-

### Mindfulness practice and emotion regulation

A one-way ANOVA revealed that there was a significant difference between groups for total adaptive regulation score (CERQ Adaptive), *F*(2, 566) = 9.81, *p* < 0.001, *η^2^* = 0.02 (Table 3). *Post hoc* tests showed that participants in the advanced practitioners group reported significantly higher use of adaptive emotion regulation (*M* = 66.9) than non-practitioners (*M* = 59.9; *p* = 0.004). This result was similar for each adaptive emotion regulation sub-dimension (Table 4).

**Table 4:** Comparison of emotion regulation strategies according to mindfulness experience group.

	NP-Non practitioners (*N* = 405)	BE-Beginners (*N* = 87)	AD-Advanced (*N* = 77)	*F*	*p* value	η^2^	*Post hoc* test (Tukey)
	Mean ± SD	Mean ± SD	Mean ± SD				
**CERQ adaptive**							
Acceptance	12.7 ± 3.81	13.1 ± 3.32	14.0 ± 3.34	4.02	0.018	0.014	NP < AD
Positive refocusing	9.60 ± 4.06	10.1 ± 3.50	11.1 ± 4.56	4.84	0.008	0.016	NP < AD
Refocus on planning	12.6 ± 3.94	13.3 ± 3.55	14.1 ± 3.60	5.97	0.002	0.020	NP < AD
Positive reappraisal	11.9 ± 4.06	12.6 ± 4.01	13.8 ± 4.19	7.04	<0.001	0.024	NP < AD
Putting into prospective	12.8 ± 3.81	13.1 ± 3.82	14.3 ± 3.46	5.13	0.006	0.017	NP < AD
**CERQ maladaptive**							
Self-blame	9.41 ± 3.92	9.85 ± 3.78	8.99 ± 3.24	1.05	0.35	0.003	
Rumination	11.9 ± 4.19	13.5 ± 4.12	11.8 ± 3.68	5.74	0.003	0.019	BE > NP, BE > AD
Catastrophizing	7.52 ± 3.40	7.54 ± 3.00	6.95 ± 2.85	1.04	0.35	0.003	
Other-blame	7.66 ± 3.59	7.49 ± 2.79	7.31 ± 2.66	0.42	0.65	0.001	

No difference among the three groups was found in the total score of maladaptive emotion regulation (CERQ Maladaptive), *F*(2,566) = 2.27, *p* = 0.10 (Table 3). Interestingly, there was only a significant difference between the three groups when looking at rumination sub-scale [*F*(2, 566) = 5.74, *p* = 0.003, *η^2^* = 0.019; Table 4]. The total score of the subscale Rumination was significantly higher in the group of beginners (*M* = 13.5) than in non-practitioners (*M* = 11.9; *p* = 0.006) and advanced practitioners (*M* = 11.8; *p* = 0.02). No differences have been found for other maladaptive subscales.

## Discussion

To our knowledge this is the first study investigating the effects of mindfulness practice on somatic symptoms and psychological distress during COVID-19 pandemic. Based on previous literature on the positive effects of mindfulness on mental health, it was assumed that dispositional mindfulness would have a buffering effect against somatic complains and the distressing mental health outcomes of COVID-19. In particular, we hypothesized that a longer experience in mindfulness practice, enhancing dispositional mindfulness, and emotion regulation, would be associated with less somatic symptoms and psychological distress. On the contrary, a lack of mindfulness experience, would bring subjects to experience more somatic symptoms and a greater use of maladaptive emotion regulation strategies that those with more experience.

### Dispositional mindfulness, somatic symptoms, and psychological distress during COVID-19 pandemic

As expected, dispositional mindfulness was associated with lower levels of somatization and psychological distress, confirming the positive relationship between this personality trait and psychological health (Tomlinson et al., 2018). A negative association has also been found with the use of maladaptive emotion regulation and with more specific COVID-19-related anxiety, suggesting that dispositional mindfulness may have afforded protective buffering against the distress caused by the pandemic. These results are in line with other studies assessing dispositional mindfulness in different populations during COVID-19 and provide new and strong evidence for the protective role of this personality trait during the pandemic (Conversano et al., 2020; Roemer et al., 2021; O’Connor et al., 2022).

### The relationship between dispositional mindfulness and mindfulness experience

In the present study, no relationships were found between dispositional mindfulness measured with MAAS (Brown and Ryan, 2003) and any of the mindfulness meditation practice factors investigated (e.g., practitioners vs. non-practitioners, years of mindfulness experience, frequency and length of practice, and type of practice). These results provide further evidence in the discrepancy between studies linking mindfulness meditation experience to higher dispositional mindfulness (Cordon and Finney, 2008; Josefsson et al., 2011; Schlosser et al., 2020), and those finding no significant associations (MacKillop and Anderson, 2007). As discussed in the study of MacKillop and Anderson (2007), in which 65% of the experienced group had practiced for 1 year or less, this might be due to the fact that the original validation of the MAAS used a sample of extensively experienced and committed Zen Buddhist practitioners (Brown and Ryan, 2003). In our population, advanced practitioners had more than 2 years of experience and cannot, therefore, be categorized as novice practitioner. However, beginner and advanced practitioner did no differ in terms of number of times practicing per week and length of practice per session. Moreover, advanced practitioners deeply involved in practicing meditation were the minority (27.3% of advanced practitioner meditated more than three times per week and only 19.5% for more than 30 min). As such, these data suggest that there are no clear differences in dispositional mindfulness (as measured by the MAAS) between beginners and advanced practitioners and also suggest that caution is needed when assessing how “regular” mindfulness meditation training has to be to modify the trait of dispositional mindfulness.

### The effect of mindfulness practice experience on somatic symptoms and psychological distress

The second hypothesis of this study, which assumed that a longer mindfulness meditation experience, enhancing dispositional mindfulness and emotion regulation, would positively affect somatic complains and psychological distress, was only partially validated. In fact, as previously noted, advanced practitioners did not show higher levels of dispositional mindfulness. Moreover, the means of somatic symptoms, COVID-19-related anxiety and psychological distress were not significantly smaller in advanced practitioners compared to the rest of the sample. However, advanced practitioners showed significantly higher use of adaptive emotion regulation strategies compared to non-practitioners, confirming the fact that emotion regulation can be considered as one of the processes facilitated by mindfulness meditation (Tang et al., 2015; Roemer et al., 2021).

The most interesting result of this study concerned the third hypothesis enunciated, for which less experienced practitioners would show more somatic symptoms and a greater use of maladaptive emotion regulation than experienced practitioners. Subjects with less experience in mindfulness practices (mindfulness experience ≤2 years) showed worse somatic health than subject who never practiced mindfulness meditation. Considering that the three groups did not differ in terms of anxiety, depression, and COVID-19-related anxiety, the higher levels of somatic symptoms in beginners cannot be explained by psychological distress. A possible explanation for this result lies in the contribution of interoception in these practices and in the possible impact of rumination, which was significantly higher in the beginners’ group. Most meditation practices, using the sensations of the body as an anchor to improve attention to the present moment, enhance interoceptive awareness and perceptual skills (Gibson, 2019). However, previous literature has highlighted the existence of two different styles of interoceptive awareness, one maladaptive and characterized by hypervigilance and catastrophizing, and one beneficial and characterized by attention regulation and acceptance (Mehling, 2016; Hanley et al., 2017; Gibson, 2019). The main difference between these two styles of interoceptive awareness is the cognitive appraisal of body states (Farb et al., 2015). Mindfulness, through acceptance, changes the appraisal and the affective significance of body states and enhances the regulation of associated negative emotions (Lindsay and Creswell, 2017; Kober et al., 2019). However, in mindfulness meditation, practitioners use interoceptive awareness since the very beginning of their practice, and their perceptual skills may improve before the competences of acceptance (Lindsay and Creswell, 2017). Thus, in distressing contexts (such as COVID-19 pandemic), an augmented attention to the body in the absence of acceptance might narrow the focus on negative stimuli and sensations, and result in more ruminative thinking (Lindsay and Creswell, 2017). Consequently, the significantly higher report of somatic symptoms in beginner practitioners could be interpreted as a consequence of the focus of attention on the body during mindfulness meditation, joined with the lack of mindful acceptance and an overuse of rumination.

## Limitations

The current study had several limitations that merit discussion. First, the convenience sampling recruitment method used does not allow any generalizations. Even though we controlled sociodemographic differences between practitioners and non-practitioners, there is still the possibility of having under/over-represented the population. Women in the sample were overrepresented (90%) not allowing gender comparisons. Second, the prevalence of somatic symptoms (56%), anxiety (36%), and depression (46%) in this sample were high compared to other studies done during the COVID-19 pandemic (Ran et al., 2020; Salari et al., 2020; Xiong et al., 2020). At the time of data collection for this study, the French population was slowly coming out of the second lockdown, which officially stopped on December 15, 2020. However, many public health measures and restrictions were put in place to continue limiting the spread of the virus. Thus, the high prevalence of somatic symptoms and psychological distress could be either a bias of the convenience sampling used in this study or the manifestation of the distress for the uncertainty of COVID-19 pandemic. Third, several factors of mindfulness practice have been assessed and the data showed significant differences between beginners and advanced practitioners in the practice of yoga, mindfulness meditation, and breathing exercises. However, previous literature has categorized specific styles of meditation (e.g., attentional, constructive, and deconstructive) based on their primary cognitive mechanisms (Dahl et al., 2015). The use of these categories could have brought a deeper understanding of the processes between mindfulness practice and somatization. Finally, we have concentrated on emotion regulation strategies to explain differences between groups as the study did not include a measure of interoceptive awareness. Therefore, we lack perspective on the possible effect of interoceptive awareness in the results, which can only be hypothesized.

## Conclusion

In conclusion, the findings of this study suggest that mindfulness practice over time enhances the use of adaptive emotion regulation. However, these practices require adequate training. Inexperience, in distressing times such as COVID-19 pandemic, can bring people to focus on bodily sensations without having developed the necessary skills to handle emerging emotions and, as a result, develop more somatic symptoms. A particular attention should be given on rumination tendencies during the initial period of mindfulness training, which is prevalent in subjects with lower mindfulness experience. Further studies are recommended to better understand the relationship between somatization, mindfulness practice, and emotion regulation and to clarify the relationship between mindfulness practice and dispositional mindfulness.

## Data availability statement

The raw data supporting the conclusions of this article will be made available by the authors, without undue reservation.

## Ethics statement

The study involving human participants was reviewed and approved by Institutional Review Board of the Laboratory of Psychology (EA4139) of the University of Bordeaux, France (protocol code: 2022.03.CLE003). The participants provided their informed consent to participate in this study online.

## Author contributions

NM, CD, and PP conceived the assessment. NM contributed to the data analysis. MB-C contributed to the data interpretation. NM, CD, MB-C, and PP participated in the concept and writing of this manuscript. All authors contributed to the article and approved the submitted version.

## Conflict of interest

The authors declare that the research was conducted in the absence of any commercial or financial relationships that could be construed as a potential conflict of interest.

## Publisher’s note

All claims expressed in this article are solely those of the authors and do not necessarily represent those of their affiliated organizations, or those of the publisher, the editors and the reviewers. Any product that may be evaluated in this article, or claim that may be made by its manufacturer, is not guaranteed or endorsed by the publisher.
