# Supplementary material for: Does the practice of mindfulness reduce somatic symptoms and COVID-19-related anxiety? A community-based survey
Source: Front Psychol. 2022 Dec 9;13:996559. doi: 10.3389/fpsyg.2022.996559 (PMC9784913; doi:10.3389/fpsyg.2022.996559)
Supplement: Supplementary file 1 [file Data_Sheet_1.docx]

Supplementary Material

# Supplementary Data

## Supplementary Table 1. Descriptive statistics of respondents’ sociodemographic characteristics and differences between practitioners and non-practitioners.

|  | Overall (N= 569) | Non-practitioners (N= 405) | Practitioners (N= 164) | *X^2^* | p-value | *V* |
| --- | --- | --- | --- | --- | --- | --- |
| **Gender** |  |  |  |  |  |  |
| Male | 53 (9.3%) | 42 (10.4%) | 11 (6.7%) | 2.667 | 0.26 | 0.068 |
| Female | 512 (90.0%) | 361 (89.1%) | 151 (92.1%) |  |  |  |
| Other | 4 (0.7%) | 2 (0.5%) | 2 (1.2%) |  |  |  |
| **Educational level** |  |  |  |  |  |  |
| Elementary or some secondary education | 55 (9.7%) | 48 (11.9%) | 7 (4.3%) | 8.205 | 0.01 | 0.120 |
| High school | 205 (36.0%) | 146 (35.8%) | 59 (36%) |  |  |  |
| Higher level of education | 309 (54.3%) | 211 (52.1%) | 98 (59.8%) |  |  |  |
| **Employment status** |  |  |  |  |  |  |
| Employed | 344 (60.5%) | 249 (61.5%) | 95 (57.9%) | 5.305 | 0.25 | 0.096 |
| Retired | 46 (8.1%) | 28 (6.9%) | 18 (11.0%) |  |  |  |
| Not active or looking for a job | 41 (7.2%) | 27 (6.7%) | 14 (8.5%) |  |  |  |
| Student | 98 (17.2%) | 75 (18.5%) | 23 (14.0%) |  |  |  |
| Other | 40 (7.0%) | 26 (6.4%) | 14 (8.5%) |  |  |  |
| **Working situation** |  |  |  |  |  |  |
| Working from home | 90 (15.8%) | 72 (17.8%) | 18 (11.0%) | 9.128 | 0.10 | 0.126 |
| Partial unemployment | 25 (4.4%) | 17 (4.2%) | 8 (4.9%) |  |  |  |
| Going to the workplace | 193 (33.9%) | 133 (32.8%) | 60 (36.6%) |  |  |  |
| Lost the employment | 39 (6.9%) | 28 (6.9%) | 11 (6.7%) |  |  |  |
| Not working | 12 (2.1%) | 150 (37.0%) | 60 (36.6%) |  |  |  |
| Other | 210 (36.9%) | 5 (1.2%) | 7 (4.3%) |  |  |  |
| **Living arrangements** |  |  |  |  |  |  |
| Alone | 98 (17.2%) | 66 (16.3%) | 32 (19.5%) | 2.135 | 0.54 | 0.061 |
| Family or partner | 446 (78.4%) | 322 (79.5%) | 124 (75.6%) |  |  |  |
| Flatmates | 23 (4.0%) | 15 (3.7%) | 8 (4.9%) |  |  |  |
| Other | 2 (0.4%) | 2 (0.5%) | 0 (0%) |  |  |  |
| **Contracted Covid** |  |  |  |  |  |  |
| Yes | 139 (24.4%) | 87 (21.5%) | 41 (25.0%) | 0.106 | 0.74 | 0.014 |
| No | 430 (75.6%) | 287 (70.9%) | 123 (75.0%) |  |  |  |
| **Positive Covid cases among relatives and close friends** |  |  |  |  |  |  |
| Yes | 282 (49.6%) | 183 (45.2%) | 84 (51.2%) | 0.156 | 0.69 | 0.017 |
| No | 287 (50.4%) | 191 (47.2%) | 80 (48.8%) |  |  |  |
| **Self-reported psychological symptoms** |  |  |  |  |  |  |
| Yes | 100 (17.6%) | 53 (13.1%) | 34 (20.7%) | 3.152 | 0.07 | 0.076 |
| No | 469 (82.4%) | 321 (79.3%) | 130 (79.3%) |  |  |  |
| **Chronic Illness** |  |  |  |  |  |  |
| Yes | 142 (25.0%) | 87 (21.5%) | 50 (30.5%) | 2.767 | 0.09 | 0.071 |
| No | 427 (75.0%) | 287 (70.9%) | 114 (69.5%) |  |  |  |
|  | Overall (N= 569) | Non-practitioners (N= 405) | Practitioners (N= 164) | *t* | p-value |  |
| **Age** |  |  |  |  |  |  |
| Mean (SD) [Min, Max] | 39.8 (14.5) [18, 89] | 38.8 (14.2) [18, 89] | 42.3 (14.8) [18, 75] | -2.586 | 0.010 |  |
| **Monthly personal income** |  |  |  |  |  |  |
| Mean (SD) | 2310 (5060) | 2180 (3630) | 2650 (7520) | -0.767 | 0.44 |  |

# R Code

## Data preparation

##Libraries

library(readxl)
library(tidyverse)
library(dplyr)
library(table1)
library(labelled)
library(ggplot2)
library(mice)
library(ltm)
library(lsr)
library(visdat)
library(xtable)
library(sjPlot) library(effsize)

##Data import

datacvFR <- read_excel("C:/Users/Final Data Covid 3 (fr).xlsx")

##Verify Missing values

#Create data frame containing only mandatory variables for all participants
dfvi <- datacvFR %>%
 dplyr::select(CODE, sex, age, study, pro, work, salary, house, covid_anxiety, Covid, CovidClose, starts_with("CERQ_"), MAAS, PSY, MC, Covid, CovidClose, PHQ8, GAD7, PHQ_15)

#Create data frame with non-mandatory variables of the questionnaire
dfnoimp <- datacvFR %>%
 dplyr::select(CODE,'work(other)', PSYtype, PSYtype_other, PSYdiag, MCtype, MINDF, MC1_other, MINDsem, MINDtime, MINDnow, MINDans, MINDtype,
 'MINDtype_other')

#Show missing values per column
vis_miss(dfvi, sort_miss = TRUE) + theme(axis.text.x = element_text(angle = 90))

Missing values less than 8% (3.8%) in all mandatory variables for the full sample. Imputation will be done on these variables using MICE pmm (predictive mean matching).

##Multiple Imputation (MICE)

#Transform the variable ‘CODE’ into a factor
dfvi <- dfvi %>%
 mutate(CODE = as.factor(CODE))

#Impute data frame with mandatory variables
init = mice(dfvi, maxit=0)

meth = init$method
predM = init$predictorMatrix

#Removing 'CODE' as a predictor
predM[, c("CODE")]=0

#Run the multiple (m=5) imputation
set.seed(103)
imputed = mice(dfvi, method=meth, predictorMatrix=predM, m=5)

#Create a data frame after imputation
impdf <- complete(imputed)

#Merge Data frame imputed and data frame containing non-mandatory variables
dfcv <- merge(impdf,dfnoimp,by="CODE")

#Drop data frames not needed
rm(datacvFR, dfnoimp, dfvi, impdf, imputed, init, predM, meth)
datacvFR <- dfcv
rm(dfcv)

## Create a new variable dividing participants by mindfulness experience levels

#Cut the variable MINDans in mindfulness levels of experience (Beginners and Advanced)
datacvFR$MINDans <- as.numeric(datacvFR$MINDans)
datacvFR$mind_experience_levels_beg_adv <- cut(datacvFR$MINDans,
 breaks=c(-Inf, 2, +Inf),
 labels=c('1','2'))

#Transform variable in a character
datacvFR$mind_experience_levels <- as.character(datacvFR$mind_experience_levels_beg_adv)

#Replace missing values in column mind_experience_levels with "3" (Non-Practitioners)
datacvFR$mind_experience_levels <- datacvFR$mind_experience_levels %>%
 tidyr::replace_na('3')

#Transform the variable in a factor with 3 levels including Non-Practitioners, Beginners and Advanced practitioners
datacvFR$mind_experience_levels <- as.factor(datacvFR$mind_experience_levels)

## Table 1. Associations between Covid-19-related factors, somatic and psychological variables

#Prepare data
cor_covid_anxiety <- datacvFR %>%
 dplyr::select(covid_anxiety, MC, PSY, Covid, CovidClose, PHQ_15, CERQ_Unadaptive, CERQ_Adaptive, PHQ8, GAD7, MAAS) %>%
 dplyr::rename(Health_issues = `MC`, Psychological_issues = `PSY`, Covid_proxy = `CovidClose`, Covid_anxiety = `covid_anxiety`)

cor_covid_anxiety[] <- lapply(cor_covid_anxiety, function(x) as.numeric(as.character(x)))
mcor2<-round(cor(cor_covid_anxiety),2)

#Print correlation table
tab_corr(cor_covid_anxiety,p.numeric=TRUE, triangle = "lower")

## Table 2. Descriptive statistics of responders’ psychological characteristics in the population (n = 569) and comparison between groups.

#p-value functions for tables

## p-value function (without the option simulate.p.value = TRUE)
pvalue <- function(x, ...) {
 # Construct vectors of data y, and groups (strata) g
 y <- unlist(x)
 g <- factor(rep(1:length(x), times=sapply(x, length)))
 if (is.numeric(y)) {
 # For numeric variables, perform a standard 2-sample t-test
 p <- t.test(y ~ g)$p.value
 } else {
 # For categorical variables, perform a chi-squared test of independence
 p <- chisq.test(table(y, g))$p.value
 }
 # Format the p-value, using an HTML entity for the less-than sign.
 # The initial empty string places the output on the line below the variable label.
 c("", sub("<", "&lt;", format.pval(p, digits=3, eps=0.001)))
}

pvalueANOVA <- function(x, ...) {
 # Construct vectors of data y, and groups (strata) g
 y <- unlist(x)
 g <- factor(rep(1:length(x), times=sapply(x, length)))

 if (is.numeric(y)) {
 # For numeric variables, perform a standard 2-sample t-test
 ano <- aov(y ~ g)
 p <- summary(ano)[[1]][[5]][1]

 } else {
 # For categorical variables, perform a chi-squared test of independence
 p <- chisq.test(table(y, g), simulate.p.value = TRUE)$p.value
 }
 # Format the p-value, using an HTML entity for the less-than sign.
 c("", sub("<", "&lt;", format.pval(p, digits=3, eps=0.001)))
}

#Prepare data for Table 2

#Prepare cut-off of variables (GAD-7, PHQ-8, PHQ-15)
datacvFR$PHQ8cut <- cut(datacvFR$PHQ8, breaks = c(-Inf,4,9,14,19,Inf),
 labels = c("PHQ-8 score 0-4 (none)", "PHQ-8 score 5-9(mild)", "PHQ-8 score 10-14(moderate)", "PHQ-8 score 15-19(moderate severe)", "PHQ-8 score 20-24(severe)"))

datacvFR$PHQ8cutoff <-cut(datacvFR$PHQ8, breaks = c(-Inf,9,+Inf),
 labels = c("PHQ-8 score < 10", "PHQ-8 score > 10"))
datacvFR$GAD7cutoff <- cut(datacvFR$GAD7, breaks = c(-Inf,9,+Inf),
 labels = c("GAD-7 score < 10", "GAD-7 score > 10"))

datacvFR$PHQ15cutoff <- cut(datacvFR$PHQ_15, breaks = c(-Inf,4,9,14,+Inf),
 labels = c("no_som","low", "medium", "high "))

datacvFR$Depression_anxiety = ifelse((datacvFR$PHQ8cutoff == "PHQ-8 score > 10") & (datacvFR$GAD7cutoff== "GAD-7 score > 10"), "yes", "no")

datacvFR$PHQ15cutoff10 <- cut(datacvFR$PHQ_15, breaks = c(-Inf,9,+Inf),
 labels = c("PHQ_15 score < 10", "PHQ_15 score > 10"))

#Data preparation for table
psy_table = datacvFR %>%
 dplyr::select(covid_anxiety,PHQ_15,PHQ15cutoff,PHQ15cutoff10,PHQ8,PHQ8cut,PHQ8cutoff,GAD7,GAD7cutoff,Depression_anxiety,MINDF, CERQ_Adaptive, CERQ_Unadaptive, MAAS, mind_experience_levels) %>%
 mutate(MINDF = recode_factor(MINDF,
 `0` = "Non practitioners",
 `1` = "Practitioners"),
 mind_experience_levels = recode_factor(mind_experience_levels,
 `3` = "Non practitioners",
 `1` = "Beginners",
 `2` = "Advanced"))

#Changing labels of the table for printing
labels <- list(
 variables=list(
 covid_anxiety="Covid-19 anxiety",
 PHQ_15= "Somatic symptoms (PHQ-15)",
 PHQ15cutoff= "PHQ-15 cut-off",
 PHQ15cutoff10="PHQ_15 cut-off score of 10",
 PHQ8= "PHQ8",
 PHQ8cut= "PHQ8 cut-off",
 PHQ8cutoff= "PHQ8 cut-off score of 10",
 GAD7="GAD-7",
 GAD7cutoff="GAD-7 cut-off",
 Depression_anxiety ="Depression_and_anxiety",
 CERQ_Adaptive="CERQ Adaptve",
 CERQ_Unadaptive= "CERQ Maladaptive",
 MAAS= "MAAS"),
 groups=list("", "", "Practitioners"))

strata <- c(list(Total=psy_table), split(psy_table, psy_table$mind_experience_levels))

#Functions for table
my.render.cont <- function(x) {
 with(stats.apply.rounding(stats.default(x), digits=3), c("",
 "Mean (SD)"=sprintf("%s (&plusmn; %s)", MEAN, SD)))
}
my.render.cat <- function(x) {
 c("",sapply(stats.default(x), function(y) with(y,
 sprintf("%d (%0.0f %%)", FREQ, PCT))))
}

#Print table
table1::table1(strata, labels, groupspan=c(1, 1, 3),
 render.continuous=my.render.cont, render.categorical=my.render.cat, extra.col=list(`P-value`=pvalueANOVA))

##Eta square for all variables
#Covid-19-related anxiety
m1 <- aov(covid_anxiety ~ mind_experience_levels, data = datacvFR)
etaSquared(m1)

#Somatic symptoms
m2 <- aov(PHQ_15 ~ mind_experience_levels, data = datacvFR)
etaSquared(m2)

#Depression
m3 <- aov(PHQ8 ~ mind_experience_levels, data = datacvFR)
etaSquared(m3)

#Anxiety
m4 <- aov(GAD7 ~ mind_experience_levels, data = datacvFR)
etaSquared(m4)

#CERQ_Adaptive
m5 <- aov(datacvFR$CERQ_Adaptive~ mind_experience_levels, data = datacvFR)
etaSquared(m5)

#CERQ_Maladaptive
m6 <- aov(datacvFR$CERQ_Unadaptive~ mind_experience_levels, data = datacvFR)
etaSquared(m6)

#Dispositional mindfulness
m6 <- aov(datacvFR$MAAS~ mind_experience_levels, data = datacvFR)
etaSquared(m6)

## Table 3. Descriptive statistics of mindfulness practice

#Data organization
table_mind <- datacvFR %>%
 dplyr::select(MINDsem, MINDtime, MINDans, MINDtype, mind_experience_levels_beg_adv) %>%
 mutate(MINDsem = recode_factor(MINDsem,
 `1` = "Less than once a week",
 `2` = "Once a week",
 `3` = "2-3 times/week",
 `4` = "4-5 times/week",
 `5` = "More than 6"),
 MINDtime = recode_factor( MINDtime,
 `1` = "10 min",
 `2` = "10-30 min",
 `3` = "30 min-2h",
 `4` = "More than 2h"),
 MINDans = recode_factor(MINDans,
 `1` = "Less than 1 year",
 `2` = "Between 1-2 years",
 `3` = "Between 3-4 years",
 `4` = "More than 4 years"),
 MINDtype = recode_factor(MINDtype,
 `1` = "Yoga",
 `2` = "Mindfulness meditation",
 `3` = "Breathing exercises",
 `4` = "Yoga and Meditation",
 `5` = "Meditation and breathing exercises",
 `6` = "Other practices"),
 mind_experience_levels_beg_adv = recode_factor(mind_experience_levels_beg_adv,
 `1` = "Beginners",
 `2` = "Advanced"))

#Changing labels for printing
var_label(table_mind) = list(
 MINDsem = "Meditation practice per week",
 MINDtime = "Lenght of meditation practice",
 MINDans = "Years of practice",
 MINDtype = "Type of practice")

#Print Table
table1::table1(~MINDans + MINDsem + MINDtime + MINDtype | mind_experience_levels_beg_adv,data = table_mind, render.missing = NULL, digits = 3, format.number = TRUE, overall='Total')

##Statistics for table
#Times of mindfulness practice per week
tbl1 <- table(datacvFR$MINDsem, datacvFR$mind_experience_levels_beg_adv)
cramersV(tbl1)

#Length of mindfulness practice per session
tbl2 <- table(datacvFR$MINDtime, datacvFR$mind_experience_levels_beg_adv)
cramersV(tbl2)

#Type of mindfulness practice
tbl3 <- table(datacvFR$MINDtype, datacvFR$mind_experience_levels_beg_adv)
cramersV(tbl3)

## Table 4. Comparison of emotion regulation strategies of the study participants according to mindfulness experience group.

#Data preparation

table4 <- datacvFR %>%
 dplyr::select(starts_with("CERQ"), mind_experience_levels) %>%
 mutate(mind_experience_levels = recode_factor(mind_experience_levels,
 `1` = "Beginners",
 `2` = "Advanced",
 `3` = "Non-Practitioners",))


table1::table1(~ CERQ_Acceptance + CERQ_Positive_refocusing+CERQ_Refocus_planning+CERQ_Positive_rappraisal+CERQ_prospective + CERQ_Selfblame+CERQ_Rumination+CERQ_Catastrophizing+CERQ_Other_blame| mind_experience_levels, data = table4, render.missing=NULL , digits = 3, format.number = TRUE, overall=F, extra.col=list(`P-value`=pvalueANOVA))

#Eta square for all variables
#CERQ_Acceptance
m1 <- aov(CERQ_Acceptance ~ mind_experience_levels, data = datacvFR)
etaSquared(m1)

#CERQ_Positive_refocusing
m2 <- aov(CERQ_Positive_refocusing ~ mind_experience_levels, data = datacvFR)
etaSquared(m2)

#CERQ_Refocus_planning
m3 <- aov(CERQ_Refocus_planning ~ mind_experience_levels, data = datacvFR)
etaSquared(m3)

#CERQ_Positive_rappraisal
m4 <- aov(CERQ_Positive_rappraisal ~ mind_experience_levels, data = datacvFR)
etaSquared(m4)

#CERQ_prospective
m5 <- aov(datacvFR$CERQ_prospective~ mind_experience_levels, data = datacvFR)
etaSquared(m5)

#CERQ_Selfblame
m6 <- aov(datacvFR$CERQ_Selfblame~ mind_experience_levels, data = datacvFR)
etaSquared(m6)

#CERQ_Rumination
m7 <- aov(datacvFR$CERQ_Rumination~ mind_experience_levels, data = datacvFR)
etaSquared(m7)

#CERQ_Catastrophizing
m8 <- aov(datacvFR$CERQ_Catastrophizing~ mind_experience_levels, data = datacvFR)
etaSquared(m8)

#CERQ_Other_blame
m9 <- aov(datacvFR$CERQ_Other_blame~ mind_experience_levels, data = datacvFR)
etaSquared(m9)

## Analysis of variance (ANOVA)

### ANOVA somatic symptoms (PHQ_15) and mindfulness experience levels

#1 assumption : All samples are independent and collected in >2 independent categorical groups
datacvFR$mind_experience_levels <- as.factor(datacvFR$mind_experience_levels)

#2 assumption: DV is continuous

#3 assumption: normal distribution of each group, no major outliers
##Shapiro test of normality
shapiro.test(datacvFR$PHQ_15)

#4 assumption: homogeneity of variances
## Bartlett test
bartlett.test(PHQ_15 ~ mind_experience_levels, data= datacvFR)

#ANOVA test
require(lsr)
model_PHQ15_practice <- model <- aov(PHQ_15~mind_experience_levels, data=datacvFR)
summary(model_PHQ15_practice)

#Effect size calculations for ANOVA
etaSquared(model_PHQ15_practice)

#Tukey-Kramer for multiple comparisons test
TukeyHSD(model_PHQ15_practice, conf.level=.95)

#Eta squared
etaSquared(model_PHQ15_practice)

### ANOVA of Adaptive emotion regulation (CERQ_Adaptive) and mindfulness experience levels

#1 assumption : All samples are independent and collected in >2 independent categorical groups
datacvFR$mind_experience_levels <- as.factor(datacvFR$mind_experience_levels)

#2 assumption: DV is continuous

#3 assumption: normal distribution of each group, no major outliers
##Shapiro test of normality
shapiro.test(datacvFR$CERQ_Adaptive)

#4 assumption: homogeneity of variances
## Bartlett test
bartlett.test(CERQ_Adaptive ~ mind_experience_levels, data= datacvFR)

#ANOVA test
require(lsr)
model_CERQ_Adaptive_practice <- model <- aov(CERQ_Adaptive ~ mind_experience_levels, data=datacvFR)
summary(model_CERQ_Adaptive_practice)

#Effect size calculations for ANOVA
etaSquared(model_PHQ15_practice)

#Tukey-Kramer for multiple comparisons test
TukeyHSD(model_CERQ_Adaptive_practice, conf.level=.95)

#Eta squared
etaSquared(model_CERQ_Adaptive_practice)

### ANOVA dispositional mindfulness (MAAS) and mindfulness experience levels (3 groups: Non-practitioners, Beginners and Advanced)

#1 assumption: all samples are independent and collected in >2 independent categorical groups
datacvFR$mind_experience_levels <- as.factor(datacvFR$mind_experience_levels)
#2 assumption: DV is continuous
#3 assumption: normal distribution of each group, no major outliers
##Shapiro test of normality
shapiro.test(datacvFR$MAAS)

#4 assumption: homogeneity of variances
#Bartlett test
bartlett.test(MAAS ~ mind_experience_levels, data= datacvFR)

#ANOVA
model_MAAS_practice <- model <- aov(MAAS~mind_experience_levels, data=datacvFR)
summary(model_MAAS_practice)

### Dispositional mindfulness (MAAS) scores and characteristics of mindfulness practice

- MAAS scores between beginner and advanced practitioners

#ANOVA
model_MAAS_practice_years = lm(MAAS ~ factor(mind_experience_levels_beg_adv), data = datacvFR)
anova(model_MAAS_practice_years)

- MAAS scores – years of mindfulness experience

#ANOVA
model_MAAS_practice_years = lm(MAAS ~ factor(MINDans), data = datacvFR)
anova(model_MAAS_practice_years)

- MAAS scores – number of times practicing per week

#ANOVA
model_MAAS_practice_times_weeks = lm(MAAS ~ factor(MINDsem), data = datacvFR)
anova(model_MAAS_practice_times_weeks)

- MAAS scores – length of practice per session

model_MAAS_practice_lenght = lm(MAAS ~ factor(MINDtime), data = datacvFR)
anova(model_MAAS_practice_lenght)

- MAAS scores – different types of practice

datacvFR <- datacvFR %>%
 mutate(MINDtype = recode_factor(MINDtype,
 `1` = "Yoga",
 `2` = "MindfulnessM",
 `3` = "Breathing ",
 `4` = "Yoga&Meditation",
 `5` = "Meditation&breathing",
 `6` = "Other"))

model_MAAS_practice_type = lm(MAAS ~ factor(MINDtype), data = datacvFR)
anova(model_MAAS_practice_type)

- Correlation between practicing more less or equally in the last month and MAAS scores

##Transforming MINDnow in a numeric continuous variable
datacvFR$MINDnow <- as.numeric(datacvFR$MINDnow)

##Correlation test
res <- cor.test(datacvFR$MAAS, datacvFR$MINDnow, use= "complete.obs")
res

## Supplementary materials table - Descriptive statistics of respondents’ Sociodemographic characteristics and differences between practitioners and non-practitioners.

#Prepare data
sociodemograpich_table = datacvFR %>%
 dplyr::select(age, sex, study, pro, work, salary, house, Covid, CovidClose, PSY, MC, MINDF) %>%
 mutate(MINDF = recode_factor(MINDF,
 `0` = "Non practitioners",
 `1` = "Practitioners"),
 sex = recode_factor(sex,
 `1` = "Male",
 `2` = "Female",
 `3` = "Other"),
 study = recode_factor(study,
 `1` = "Elementary or some secondary education",
 `2` = "Highschool",
 `3` = "Higher level of education"),
 pro = recode_factor(pro,
 `1` = "Employed",
 `2` = "Retired",
 `3` = "Not active or looking for a job",
 `4` = "Student",
 `5` = "Other"),
 house = recode_factor(house,
 `1` = "Alone",
 `2` = "Family or partner",
 `3` = "Flatmates",
 `4` = "Other"),
 work = recode_factor(work,
 `1` = "Working from home",
 `2` = "Partial unemployment",
 `3` = "Going to the Workplace",
 `4` = "Lost the employment",
 `5` = "Other",
 `6` = "Not working"),
 PSY = recode_factor(PSY,
 `1` = "Yess",
 `0` = "No"),
 MC = recode_factor(MC,
 `1` = "Yess",
 `0` = "No"),
 Covid = recode_factor(Covid,
 `1` = "Yess",
 `0` = "No"),
 CovidClose = recode_factor(CovidClose,
 `1` = "Yess",
 `0` = "No"))

#Change labels for printing
labels <- list(
 variables=list(
 age = "Age",
 sex = "Gender",
 study = "Educational level",
 pro = "Employment status",
 work = "Working situation",
 house = "Living arrangements",
 salary="Monthly personal income",
 Covid="Contracted Covid",
 CovidClose="Positive Covid cases among relatives and close friends",
 PSY="Psychological problems",
 MC="Chronic Illness"))

#Print Table
table1::table1(~age +sex+study+pro+work+salary+house+Covid+CovidClose+PSY+MC|MINDF, data = sociodemograpich_table, render.missing=NULL , digits = 3, format.number = TRUE, overall=T)

#Statistics for tables
chisq.test(table(datacvFR$sex, datacvFR$MINDF))

chisq.test(table(datacvFR$study, datacvFR$MINDF))

chisq.test(table(datacvFR$pro, datacvFR$MINDF))

chisq.test(table(datacvFR$work, datacvFR$MINDF))

chisq.test(table(datacvFR$house, datacvFR$MINDF))

chisq.test(table(datacvFR$Covid, datacvFR$MINDF))

chisq.test(table(datacvFR$CovidClose, datacvFR$MINDF))

chisq.test(table(datacvFR$PSY, datacvFR$MINDF))

chisq.test(table(datacvFR$MC, datacvFR$MINDF))

t.test(age~MINDF,data=datacvFR)

cohen.d(datacvFR$age,datacvFR$MINDF,pooled=TRUE,paired=FALSE, na.rm=TRUE)

t.test(salary~MINDF,data=datacvFR)

cohen.d(datacvFR$salary,datacvFR$MINDF,pooled=TRUE,paired=FALSE,na.rm=TRUE)
